# Impact of the 2014–2016 El Niño on Geohelminth Control in Stray Dogs and Cats Without Anthelmintic Treatment

**DOI:** 10.1155/vmi/9294504

**Published:** 2026-07-11

**Authors:** Prasit Na-Ek, Attarat Pattanawongsa, Patthanasak Khammaneechan, Blego Sedionoto, Witthaya Anamnart

**Affiliations:** ^1^ Department of Medical Science, School of Medicine, Walailak University, Nakhon Si Thammarat, 80161, Thailand, wu.ac.th; ^2^ Research Center in Tropical Pathobiology, Walailak University, Nakhon Si Thammarat, 80161, Thailand, wu.ac.th; ^3^ School of Pharmacy, Walailak University, Nakhon Si Thammarat, 80161, Thailand, wu.ac.th; ^4^ Drug and Cosmetics Excellence Center, Walailak University, Nakhon Si Thammarat, 80161, Thailand, wu.ac.th; ^5^ School of Public Health, Walailak University, Nakhon Si Thammarat, 80161, Thailand, wu.ac.th; ^6^ EC for PHR, Walailak University, Nakhon Si Thammarat, 80161, Thailand, wu.ac.th; ^7^ Department of Environmental Health, Faculty of Public Health, Mulawarman University, Samarinda, East Kalimantan, Indonesia, unmul.ac.id; ^8^ School of Allied Health Sciences, Walailak University, Nakhon Si Thammarat, 80161, Thailand, wu.ac.th

**Keywords:** a stray cat, a stray dog, drought, El Niño 2014–2016, soil-transmitted helminthiases

## Abstract

**Background:**

Soil‐transmitted helminthiases (STHs) in stray dogs and cats, including hookworm infection, strongyloidiasis, and toxocariasis, are prevalent globally. The lack of STH control in these stray animals leads to an increased prevalence of STHs in southern Thailand. Suddenly, the El Niño 2014–2016 induced a 14‐month‐long drought. We thus aimed to compare the prevalence of STHs in stray cats in artificial wet sandy soil (unaffected area) and dry sandy soil (affected area) before and after drought. Furthermore, we also observed the prevalence of STHs in stray dogs in another study area before and after drought.

**Methods:**

A comparative study was conducted on 77 stray cats, 17 in the unaffected area and 60 in the affected area. Stools were collected before (2014) and after the drought (2016 and 2020). In study area 2, the respective 122 and 131 stools were collected from stray dogs before (2014) and after the drought (2020). STHs were detected using a modified formalin‐ether concentration technique, agar plate culture, and 1:20 water‐diluted stool suspension. Rainfall and rainy‐day data, serving as drought indicators, were retrieved from the two nearest local meteorological stations.

**Results:**

In stray cats, the prevalence of STHs in the unaffected area remained unchanged pre‐ and postdrought. In the affected area, the prevalence of hookworm infection, but not strongyloidiasis and toxocariasis, decreased after drought. However, over time, the unchanged prevalence of strongyloidiasis in 2016 decreased in 2020 compared to that in 2014. Furthermore, the prevalence of hookworm infection in stray dogs in study area 2 decreased after the drought, while eliminating strongyloidiasis and toxocariasis. Notably, the prevalence of *Spirometra* spp. infection in stray dogs and in cats in the unaffected area remained unchanged.

**Conclusion:**

El Niño 2014–2016‐induced drought, one of the climatic factors, demonstrates the significant impact of climatic factors on STH transmission in stray animal populations where anthelmintic treatment is not applied, highlighting the need to integrate climate forecasting into future control strategies.

## 1. Introduction

Dog hookworms, including *Ancylostoma caninum*, *Ancylostoma braziliense*, *Ancylostoma ceylanicum*, *Ancylostoma tubaeforme*, and *Uncinaria stenocephala*, and cat hookworms, including all except for the first species, cause anemia in dogs and cats [1]. Dogs and cats are reservoirs of *A. ceylanicum*, one of the human hookworms [2]. These *Ancylostoma* spp., except for *A. ceylanicum*, cause cutaneous larva migrans in humans [3]. *Toxocara canis* and *Toxocara cati* are dog and cat roundworms, respectively. *T. canis* and *T. cati* cause toxocariasis in dogs and cats. Both species can cause human toxocariasis, including visceral larva migrans, ocular larva migrans, neurotoxocariasis, and common or covert toxocariasis [4]. *Strongyloides* spp. cause diarrhea in infected dogs and cats [5]. In addition, dogs and cats are reservoirs of *S. stercoralis* [6, 7], which causes human strongyloidiasis, a fatal disease in immunosuppressed patients [8]. These animal soil‐transmitted helminthiases (STHs) are prevalent worldwide in tropical and nontropical regions [9–12]. *Spirometra* spp. is a cestode residing in the host intestine. Dogs and cats are the definitive hosts, copepods are the first intermediate hosts, and the second intermediate hosts are tadpoles and frogs. Snakes and birds can be paratenic hosts [13].

More than 700 million stray dogs and cats live in human communities globally [14]. In 2016, there were approximately 758,446 stray dogs and 474,142 stray cats all over Thailand [15]. These stray animals are infected with helminths, including STHs [16]. Similarly, STHs, including hookworm infection, strongyloidiasis, and toxocariasis, are highly prevalent in stray dogs and cats living in southern Thailand due to the tropical rainforest and tropical monsoon climate, characterized by a long rainy season and a short dry season [17]. Annual rainfall is more than 2400 mm [18]. Rainfall‐induced moist soil maintains the life cycle of STHs in humans and animals [19].

From 2012 to 2014, we attempted to control human STHs in an Islamic village located on the seashore where trichuriasis is predominantly prevalent. The prevalence of human trichuriasis in artificial moist soil was higher than in the area with dry sandy soil (100%–35%). We suspected that stray cats in the wet soil area might be a reservoir of *Trichuris* spp. We collected stools from stray cats in moist and dry soil areas and examined them using a modified formalin‐ether concentration technique (MFECT). Unfortunately, in the wet soil area, we found no eggs of *Trichuris* spp. However, hookworm eggs, *T. cati* eggs, and tapeworm *Spirometra* spp. eggs were found in 100%, 20%, and 20% of 20 stool samples, respectively. And 40% were positive for *Strongyloides* spp. using agar plate culture (APC). In addition, the results in the dry soil area are similar to those in the moist soil area except for the absence of *Spirometra* spp. These stray cats remained untreated due to the lack of control over these worms. Unexpectedly, the drought occurred from February 2014 to March 2015 [20, 21]. The 14‐month‐long drought is one of the factors contributing to a decrease in the prevalence of human STHs, including ascariasis, trichuriasis, and hookworm infection in another study area in Mokhalan Subdistrict, Thasala District, Nakhon Si Thammarat (NST), southern Thailand. Moreover, the prevalence of strongyloidiasis also decreased over the following 3 years. Other factors contributing to the reduced prevalence of human STHs are improved hygiene practices and control of human STHs in the dry season [21]. Conversely, mass drug administration and improved sanitation and hygiene practices cannot be implemented in the control of STHs in stray animals in these areas. Therefore, the data on the prevalence of STHs in stray dogs and cats before and after the drought may reveal that the 2014–2016 El Niño‐induced 14‐month‐long drought caused a decrease in the prevalence of STH in stray dogs and cats. We thus evaluated the impact of drought on the reduction in the prevalence of STHs in stray dogs and cats.

## 2. Materials and Methods

### 2.1. Study Design and Study Areas

**FIGURE 1:**
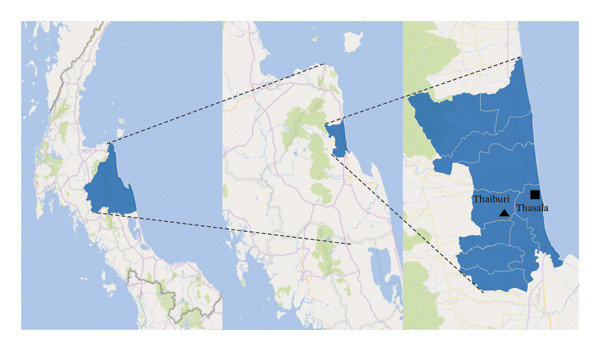
Maps of the two study areas: ■ represents study area 1, and ▲ represents study area 2, located in the respective Thasala and Thaiburi Subdistricts, Thasala District, Nakhon Si Thammarat, southern Thailand. Maps were generated using Microsoft 365 (Excel) with publicly available data sources from the Thailand GIS repository (retrieved on July 8, 2025).

From February 2014 to January 2020, we conducted a comparative study on the impact of drought on the decrease in the prevalence of cat STHs in study area 1 (Figure 1). The study area is located in Village 4, Thasala Subdistrict, Thasala District, NST, southern Thailand, 8 km from our Walailak University laboratory. The rural village is on the seashore, where all the villagers are Muslims. Thus, people raised a cat but not a dog. The unaffected area consists of three houses with roofs close together, so the sandy ground would not receive sunlight. Three families lived here. People washed their feet before entering their homes. This characteristic made the sandy soil wet all the time. Seventeen stray cats have resided in this artificial wet soil (unaffected by the drought) area. The soil in this area remained visibly moist throughout the drought due to regular foot‐washing activities and constant shading from adjacent buildings. Moreover, water retention in some sandy soil holes due to extensive use of water for foot‐washing could be frequently seen. People living here are fishermen who prepare shrimp, crab, and fish for their food. Notably, some raw parts of shrimp, crab, and fish were left in a sandy soil hole, retained with tap water. These stray cats sometimes receive some food, including raw shrimp, crab, and fish bodies, from people who live here. However, they do not raise the cats as their pets.

Conversely, the area affected by drought is the sand beach designed to attract people, where 20 small vendors sell food. The affected area is about 1 km away from the unaffected area. The affected area consisted of an 800 m‐long gravel road. Pine, coconut, and other trees have been planted on both sides of the road to provide shade. A cluster of 60 stray cats has resided here, receiving food from people. Cats around the village were excluded from the experiment, as they may receive anthelmintics from their owners. In 2014, we collected fresh stool within two hours of defecation from 60 stray cats in the affected area and 17 in the unaffected area. The follow‐up was conducted again in 2016 and 2020.

Study area 2 is in and around Walailak University, Thaiburi Subdistrict, Thasala District, NST (Figure 1). The urban area has many buildings with concrete streets. At Walailak University, there are two big reservoirs. Water from the upper reservoir is drained as follows: (a) to the lower reservoir via a man‐made canal linked between the two reservoirs, then to a natural canal in the urban area outside Walailak University and (b) to the second natural canal outside Walailak University via another man‐made canal linked between the upper reservoir and the second natural canal. Stray dogs have resided in this area. These dogs defecate on the cement surface and the sandy‐loamed soil exposed to sunlight. We collected 122 stray dog stools in 2014 and 131 stools in 2020. Dog and cat stools were processed within 2 h of defecation.

Unexpectedly, the drought started in February 2014 and ended in March 2015. There was little rainfall during the 14‐month drought. Therefore, we surveyed the prevalence of dog and cat STHs before and after the drought.

### 2.2. Laboratory Procedure

A MFECT, APC, and 1:20 water‐diluted stool suspension (SS) methods were employed to detect cat and dog STH. MFECT was used to detect eggs of *T. cati, T. canis*, hookworm, and other intestinal helminth eggs and larvae. Moreover, the APC detected *Strongyloides* spp. and hookworm third‐stage larvae (L3). In addition, SS detected *Strongyloides* spp. and hookworm L3 in cases of *Rhabditis* spp. co‐infection or contamination, as growth and development of *Rhabditis* spp. were suppressed by water when in SS. APC and SS were employed to confirm MFECT’s ability to detect hookworms, as *Rhabditis*‐related eggs can resemble those of hookworms.

The MFECT procedure was as follows [22]: 2 g of stool was suspended in 8 mL of NSS in a conical tube. The suspension was strained through 2 wire meshes: a coarse one on top and a fine one in the funnel and into another tube. The strained suspension was centrifuged at 700 × *g* for 5 min. The supernatant was then decanted. After that, the volume was adjusted to 7 mL with 10% formalin, followed by 3 mL of diethyl ether. After mixing well, the suspension was immediately centrifuged at 700 × *g* for 5 min. The debris plug was loosened and decanted. The sediment was adjusted to 0.75–1 mL (25–35 drops) with 10% formalin. Two drops were examined for the number of eggs and divided by 2. The mean eggs were multiplied by 25–35, divided by 2, and presented as the number of eggs per gram of stool. The negative results were reported after examination of the entire sediment. Hookworm, *T. cati*, and *T. canis* eggs were counted and presented as egg numbers per gram of stool. L1 of *Strongyloides* spp. and eggs of *Spirometra* spp. were reported as positive or negative. Notably, L1 of *Strongyloides* spp. has a rhabditoid esophagus, a short buccal cavity, and a distinct genital primordium. *Rhabditis* spp. L1 resembles L1 of *Strongyloides* spp. but with the long buccal cavity.

The APC method was as follows [23]: 2 g of stool was placed in the center of the nutrient agar plate. The stool was then incubated at room temperature for 6 days. The growth and development of *Strongyloides* spp., hookworm, and *Rhabditis* spp. were checked daily. After the agar surface was flushed with 10 mL of 10% formalin, the formalin‐containing larvae were transferred back to a centrifuged tube and spun at 700 × *g* for 5 min. The pellets were then examined for larvae and free‐living males and females.

The SS method was as follows [24]: 1 g of stool was added to 19 mL of distilled water in a 150 mm plastic plate and mixed well. The SS was incubated at room temperature for 6 days. We followed the growth and development of *Strongyloides* spp., hookworm, and *Rhabditis* spp. daily.

For cases of suspicion of hookworm or *Strongyloides* spp. L3, we transferred the suspected L3 to a glass slide containing a drop of 10% formalin. After exposure to formalin, the L3 of the two worms was identified at 10 and 30 min, respectively. L3 and free‐living males and females of *Strongyloides* spp. and all stages of rhabditid worms would die within 10 min, while hookworm L3 were viable and dead at 30 min or more. L3 of *Strongyloides* spp. had a long filariform esophagus about one‐half of the body length and a knotted tail, while hookworm L3 had a short filariform esophagus and a pointed tail. Some *Rhabditis* spp. can resemble *Strongyloides* spp. L3, except for the blunt tail.

### 2.3. Observation on the Growth and Development of STH Eggs or Larvae on Moist and Dry Media

Seven cat stools were collected: 3 infected with hookworm, three infected with *Strongyloides* spp., and one infected with *T. cati*. Eggs or larvae were counted using MFECT. Each gram of stool was placed on dry soil in a 35 mm dish, moist soil in a 35 mm dish, and nutrient agar in a 90 mm dish. Daily, a few drops of DW were added to moisten the stool and soil in a moist soil dish. All dishes were kept at room temperature (26°C–31°C). Relative humidity increased from 65% to 75% in the daytime to 90%–96% at night. For hookworm and *Strongyloides* spp., eggs or larvae were observed at 30, 48, and 60–80 h using a triplicate direct fecal smear. After 6 days of incubation, 3 mL of DW were added to dry soil or moist soil dishes. Viable L3 of *Strongyloides* spp. would swim to the water surface and be transferred to a new dish containing 1 mL of 10% formalin for counting. Hookworm L3, which did not swim to the water surface, was counted in the original dishes. 10 mL of 10% formalin was added to the agar surface, left for 30 min, and transferred to a conical tube. After centrifugation at 700 × *g* for 5 min, the larvae in the sediment were identified and counted. For *T. cati*, eggs were observed at 1, 2, 3, 4, 5, and 6 weeks using a triplicate direct fecal smear. After 6 weeks, eggs in stool placed on three media were counted using MFECT.

### 2.4. Rainfall, Rainy Days, Dry Spell Days, Maximum Temperature, and Relative Humidity

From 2014 to 2020, we retrieved the maximum temperature and relative humidity from the Thai Meteorological Department, Thailand (https://www.tmd.go.th). Similarly, from 2014 to 2016, rainfall and rainy‐day data in NST were retrieved from the same source. From 2014 to 2020, dry spell days, a sequence of 15 or more consecutive nonrainy days in the wet season with less than 1 mm daily, and rainfall and rainy‐day data in Thailand, the eastern coast of southern Thailand, NST, and the two meteorological stations, stations 0740 and 0748, were obtained from https://www.tmd.go.th and the National Hydroinformatics Data Center, Thailand (https://www.thaiwater.net), respectively. The two stations, 0740 and 0748, were chosen as reference stations due to their proximity and completeness of their data, located 16 km and 15 km west and south of the study areas, respectively.

### 2.5. Statistical Analysis

All statistical analyses were performed using IBM SPSS Statistics (Version 27; IBM Corp., Armonk, NY, USA), in accordance with the Statistical Analyses and Methods in the Published Literature (SAMPL) guidelines. The Shapiro–Wilk test was utilized to assess the normality of continuous data (infection intensity, measured in eggs per gram). Normally distributed continuous variables (e.g., intensity of toxocariasis) were summarized as means and standard deviations (SDs) with ranges. Non‐normally distributed continuous variables (e.g., intensity of hookworm infection in cats in the affected area and in dogs) were summarized as medians and interquartile ranges (IQRs) with ranges. Categorical variables (prevalence of infection) were presented as counts and percentages, with 95% confidence intervals (CIs) calculated using the Wilson score interval method. To compare differences in prevalence before and after the drought, we used Pearson’s chi‐square test or Fisher’s exact test, as appropriate, and reported odds ratios (ORs) with 95% CIs as measures of effect size. Differences in the intensity of infection between independent time periods were analyzed using the independent‐samples *t*‐test for normally distributed data and the Mann–Whitney *U* test for non‐normally distributed data. Cliff’s delta was calculated to report the effect size for nonparametric continuous comparisons. All statistical tests were 2‐sided, and an a priori level of significance was set at *p* < 0.05.

## 3. Results

From 2014 to 2020, the average maximum temperature in NST ranged from 34.5°C to 35.3°C. Notably, the maximum temperature from April to July in 2014 and 2015 during drought ranged from 37°C to 38°C (Table 1). The average relative humidity ranged from 81% to 85%. However, the average relative humidity decreased to 76%–79% from February to July 2014 (Table 2). From 2014 to 2020, NST experienced the maximum annual rainfall compared to provinces located on the eastern coast of southern Thailand and the whole country, respectively (Table 3).

**TABLE 1:** Maximum temperature in Nakhon Si Thammarat from 2014 to 2020.

Year	Maximum temperature (°C) during 2014–2020												
	Jan	Feb	Mar	Apr	May	Jun	Jul	Aug	Sep	Oct	Nov	Dec	Aver
2014	32.5	32.5	35.5	*37.0*	*38.0*	36.7	*37.2*	36.2	36.0	34.5	33.6	33.5	*35.3*
2015	31.6	32.4	34.9	*37.0*	*37.1*	*37.1*	*37.0*	36.2	35.1	35.0	33.2	32.9	*35.0*
2016	33.7	32.5	34.6	*38.0*	*38.1*	36.5	35.5	36.0	36.2	35.2	33.6	33.5	*35.3*
2017	32.6	33.4	35.0	35.7	35.7	36.5	35.5	35.6	35.8	35.8	33.5	32.7	34.8
2018	32.5	33.0	34.1	35.6	35.2	36.5	36.0	36.0	*37.0*	34.4	32.2	34.3	34.7
2019	32.5	33.1	36.8	*37.4*	*38.4*	*37.1*	*37.3*	*37.0*	35.5	33.9	33.1	31.8	*35.3*
2020	32.6	32.7	36.5	36.3	35.8	35.1	34.7	35.2	35.4	34.5	32.6	32.3	34.5

*Note:* The data were retrieved from https://www.tmd.go.th on June 6, 2025. Only italic values indicate commonly higher temperatures exceeding 37°C during the drought periods in 2014–2016 and 2018–2019. The average temperature was also higher than that during the La Niña period.

**TABLE 2:** Relative humidity in Nakhon Si Thammarat from 2014 to 2020.

Year	Relative humidity during 2014–2020												
	Jan	Feb	Mar	Apr	May	Jun	Jul	Aug	Sep	Oct	Nov	Dec	Aver
2014	83	*79*	*78*	*76*	80	*78*	*77*	80	83	87	89	87	81
2015	84	80	*79*	80	*79*	81	78	83	84	87	91	88	83
2016	85	83	80	*77*	80	81	84	80	81	86	90	90	83
2017	91	84	82	85	82	80	81	82	85	85	89	88	85
2018	88	81	80	82	84	81	*77*	*79*	83	87	89	89	83
2019	85	80	*78*	*77*	*78*	*78*	*78*	*77*	84	87	88	87	81
2020	83	82	*77*	81	83	84	84	82	84	87	89	88	84

*Note:* The data were retrieved from https://www.tmd.go.th on June 6, 2025. Only italic values indicate commonly lower (<80) relative humidity during the drought period in 2014–2016 and 2018–2019.

**TABLE 3:** Rainfall in 2 local meteorological stations, Nakhon Si Thammarat (NST) Province, southern part, Thailand, and dry spell length days in NST during 2014–2020.

Year	Rainfall					No. of dry spell days	
	Thailand	Southern Thailand	NST	STN 0740^a^	STN 0748^a^	NST (dry season)	NST (wet season)
2014	1520.4	1878	2318.5	89	13.5	*51*	1
2015	1419.7	1648	2091.5	1949	827.7	*31*	2
2016	1716.1	1911	2449.3	1404	696.5	*29*	4
2017	2017.1	2767	4478.4	2925.5	3924.5	17	6
2018	1660.9	1988	2218.8	2183.5	2407	11	5
2019	1343.4	1611	2132.2	774.5	1200.5	*38*	5
2020	1527.3	2154	3141.6	1478.5	792	11	ND

*Note:* STN = local meteorological station, NST = Nakhon Si Thammarat Province. Data were retrieved from https://www.thaiwater.net/weather/rain on June 20, 2025. Italic values focus on long dry‐spell days during the drought in 2014–2016 and in 2019.

Abbreviation: ND = no data.

^a^Reference station in this study.

Droughts, triggered by El Niño episodes, occurred from February 2014 to April 2015. From February–April 2014 to May–December 2014, minimal rainfall (1.6, 0, and 3 mm/month in NST; 0, 0, 2.5, 3, 24, 0, 25, and 34.5 mm/month at local meteorological station 0740; 0.5, 9.5, 0, 2, and 1.5 mm/month at local meteorological station 0748) was observed. Rainfall and rainy days in NST from February to April 2014 were the average measurements of all local meteorological stations. Monthly rainfall and rainy days at local meteorological station 0740 or station 0748 were measured only at station 0740 or station 0748. Additionally, a significant lack of rainy days continued during these periods (3 of 89 days in NST, 4 of 152 days at station 0748, and 16 of 214 days at station 0740). In 2015, little rainfall occurred from January to April (54.5, 3.5, 1.5, and 6 mm at station 0748; 139, 30.5, 7.5, and 30.5 mm at station 0740) with few rainy days (12 of 120 days at station 0748 and 20 of 120 days at station 0740) (Tables 4, 5, 6). In addition, the respective 51 and 31 dry spell days occurred in 2014 and 2015 (Table 3).

**TABLE 4:** Rainfall and rainy days in Nakhon Si Thammarat from 2014 to 2016.

Year	Monthly rainfall in mm (rainy days) during 2014–2016												
	Jan	Feb	Mar	Apr	May	Jun	Jul	Aug	Sep	Oct	Nov	Dec	Tot
2014	267.5 (12)	*1.6 (2)*	*0 (0)*	*0.3 (1)*	202.2 (13)	93.8 (11)	75.3 (16)	159.1 (17)	123.3 (21)	337.6 (22)	418.6 (21)	639.2 (24)	2318.5 (160)
2015	*69.5 (2)*	*18.3 (2)*	*0.2 (1)*	112.6 (7)	65.5 (11)	63.1 (10)	96.3 (14)	214.3 (16)	145.3 (19)	326.8 (19)	579.9 (24)	399.7 (20)	2091.5 (155)
2016	114.7 (12)	118.3 (10)	0 (0)	0 (0)	44.4 (11)	114.7 (14)	140.7 (17)	37.7 (15)	116.1 (19)	272.3 (23)	379.5 (24)	1110.9 (23)	2449.3 (168)

*Note:* The data were retrieved from https://www.tmd.go.th on June 20, 2025. Italic values indicate lower overall rainfall and fewer rainy days in Nakhon Si Thammarat during February–April 2014 and January–March 2015.

**TABLE 5:** Rainfall and rainy days were measured at station 0740.

Year	Rainfall in mm (no. of rainy days)												
	Jan	Feb	Mar	Apr	May	Jun	Jul	Aug	Sep	Oct	Nov	Dec	Tot
2014	ND	ND	ND	ND	*0 (0)*	*0 (0)*	*2.5 (1)*	*3 (1)*	*24 (3)*	*0 (0)*	*25 (8)*	*34.5 (3)*	*89 (16)*
2015	*139 (8)*	*30.5 (2)*	*7.5 (3)*	30.5 (7)	198 (12)	3.5 (3)	134 (14)	136 (15)	236 (14)	351 (16)	424 (19)	259 (11)	1949 (124)
2016	ND	ND	ND	ND	ND	ND	90.5 (9)	76.5 (17)	51.5 (19)	200 (13)	156.5 (18)	829 (15)	1404 (91)
2017	831.5 (16)	139 (12)	76.5 (5)	170 (11)	168 (14)	73.5 (11)	22 (10)	66.5 (14)	186 (13)	100 (12)	460 (14)	637 (12)	2925 (144)
2018	54.5 (13)	99.5 (6)	95 (6)	118 (8)	135 (9)	55.5 (11)	68.5 (18)	58 (10)	178 (16)	205 (18)	688 (17)	425 (18)	2184 (150)
2019	148 (13)	23.5 (5)	0 (0)	0 (0)	140 (9)	87 (8)	80 (9)	29.5 (11)	31 (8)	16.5 (2)	166 (11)	53 (7)	774.5 (83)
2020	39.5 (6)	115 (8)	3 (1)	159 (6)	36 (9)	170 (15)	145 (12)	56.5 (12)	244 (16)	162 (14)	262 (15)	86 (8)	1478 (122)

*Note:* Data were retrieved from https://www.thaiwater.net/weather/rain on June 20, 2025. Italic values showed little rainfall and fewer rainy days during May 2014–March 2015.

Abbreviation: ND = no data.

**TABLE 6:** Rainfall and rainy days were measured at station 0748.

Year	Rainfall in mm (no. of rainy days)												
	Jan	Feb	Mar	Apr	May	Jun	Jul	Aug	Sep	Oct	Nov	Dec	Tot
2014	ND	ND	ND	ND	*0.5 (1)*	*9.5 (1)*	ND	*0 (0)*	*2 (1)*	ND	*1.5 (1)*	ND	*13.5 (4)*
2015	*54.5 (6)*	*3.5 (2)*	*1 (1)*	*6 (3)*	104 (13)	80 (10)	192 (19)	229 (18)	157.7 (13)	0 (0)	0 (0)	0 (0)	827.7 (85)
2016	0 (0)	ND	ND	ND	ND	ND	0 (0)	0 (0)	0 (0)	0 (0)	74 (2)	622.5 (10)	696.5 (12)
2017	889 (12)	180 (8)	146 (8)	298 (12)	310 (20)	217 (10)	225 (14)	186 (14)	287 (16)	128.5 (11)	525 (13)	360 (10)	3924.5 (146)
2018	124 (14)	15 (7)	191 (7)	71 (8)	66 (7)	262 (14)	274 (18)	175 (15)	326 (18)	181 (22)	252 (17)	355 (15)	2407 (152)
2019	246 (8)	13 (4)	0 (0)	0 (0)	192.5 (10)	76 (9)	3.5 (3)	21 (6)	139 (6)	430 (12)	65 (9)	14.5 (4)	1200.5 (71)
2020	5 (4)	10 (3)	0.5 (1)	28 (9)	121 (11)	233.5 (13)	264 (13)	119.5 (14)	2.5 (1)	0 (0)	1.5 (2)	6.5 (1)	792 (72)

*Note:* Data were retrieved from https://www.thaiwater.net/weather/rain on June 20, 2025. Italic values showed little rainfall and fewer rainy days during May 2014–April 2015.

Abbreviation: ND = no data.

After El Niño 2014–2016, La Niña 2017–2018 occurred. The following El Niño 2018–2019, caused drought from 2019 to 2020 (Tables 5 and 6). Thirty‐eight dry spell days in 2019 indicated drought in 2019 (Table 3).

### 3.1. Effect of Drought on the Prevalence of STHs and *Spirometra* spp. Infection in Stray Cats in Affected and Unaffected Areas

**FIGURE 2:**
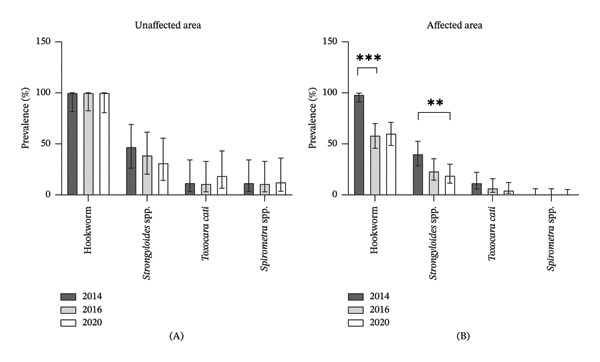
Comparative prevalence of soil‐transmitted helminthiases and *Spirometra* spp. infection in stray cats. The clustered bar charts illustrate the prevalence (%) of each helminth in (A) the unaffected area with artificial wet sandy soil and (B) the affected area with drought‐caused dry sandy soil. Data are presented across three time points: predrought (2014), postdrought (2016), and follow‐up (2020). Error bars represent 95% confidence intervals (Wilson score interval). Asterisks indicate statistically significant decreases in prevalence in the affected area compared to the 2014 predrought baseline (^∗∗∗^ *p* < 0.001 for hookworm comparing 2014 to 2016, Fisher’s exact test; ^∗∗^ *p* = 0.009 for *Strongyloides* spp. comparing 2014 to 2020, Pearson’s chi‐square test).

The prevalence of STHs in stray cats, including hookworm infection, strongyloidiasis, toxocariasis, and the cestode species *Spirometra* spp. infection, in the unaffected area remained unchanged before (2014) and after the drought (2016). In contrast, after the drought, the prevalence of hookworm infection in the affected area decreased from 98.3% (59/60; 95% CI: 91.1%–99.7%) to 58.3% (35/60; 95% CI: 45.7%–69.9%) (Fisher’s exact test: OR = 42.14, 95% CI: 5.47–324.77; *p* < 0.001). Furthermore, the intensity of hookworm infection in the affected area, which was non‐normally distributed, significantly decreased from a median of 500 (IQR: 260–840; range: 60–1360) eggs per gram to 260 (IQR: 120–520; range: 40–1020) eggs per gram (Mann–Whitney *U* test: Cliff’s delta = 0.33; *p* = 0.007). However, the prevalence of toxocariasis remained unchanged. For strongyloidiasis, although the prevalence decreased from 40.0% (24/60) in 2014 to 23.3% (14/60) in 2016, this immediate postdrought difference was not statistically significant (Fisher’s exact test, *p* = 0.077). Nevertheless, by the 2020 follow‐up, the prevalence had significantly decreased to 19.1% (13/68) compared to the predrought level (Pearson’s chi‐square test: OR = 2.82, 95% CI: 1.27–6.25; *p* = 0.009). Following the La Niña events in 2017 and 2018 and the El Niño events in 2019 and 2020, the prevalence of cat STHs in affected and unaffected areas did not show further significant changes (Figure 2 and Table S1).

### 3.2. Effect of Drought on the Prevalence of STHs and *Spirometra* spp. Infection in Stray Dogs

**FIGURE 3:**
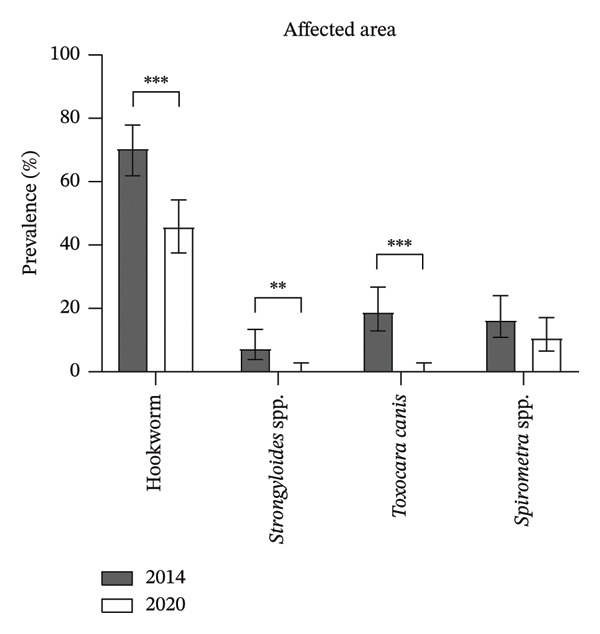
Comparative prevalence of soil‐transmitted helminthiases and *Spirometra* spp. infection in stray dogs. The clustered bar chart illustrates the prevalence of each helminth in stray dogs residing in and around Walailak University (study area 2). Data are presented for predrought (2014) and postdrought (2020) periods. Error bars represent 95% confidence intervals (Wilson score interval). Asterisks indicate a statistically significant decrease in prevalence after the drought (^∗∗^ *p* = 0.001; ^∗∗∗^ *p* < 0.001, based on Pearson’s chi‐square test for hookworm and Fisher’s exact test for *Strongyloides* spp. and *Toxocara canis*).

After the El Niño events in 2014–2015 and 2018–2019 and a La Niña event in 2017, the prevalence of hookworm infection in stray dogs in study area 2 decreased from 70.5% (86/122; 95% CI: 61.9%–77.9%) to 45.8% (60/131; 95% CI: 37.5%–54.3%) (Pearson’s chi‐square test: OR = 2.83, 95% CI: 1.68–4.75; *p* < 0.001). The intensity of hookworm infection also significantly decreased from a median of 600 (IQR: 380–940; range: 60–4680) eggs per gram to 100 (IQR: 40–310; range: 20–9800) eggs per gram (Mann–Whitney *U* test: Cliff’s delta = 0.58; *p* < 0.001). Furthermore, after the drought, strongyloidiasis and toxocariasis were eliminated. The prevalence of strongyloidiasis decreased from 7.4% (9/122; 95% CI: 3.9%–13.4%) to 0% (0/131; 95% CI: 0.0%–2.8%) (Fisher’s exact test, *p* = 0.001), and toxocariasis decreased from 18.9% (23/122; 95% CI: 12.9%–26.7%) to 0% (0/131; 95% CI: 0.0%–2.8%) (Fisher’s exact test, *p* < 0.001). No eggs of *T. canis* or larvae of *Strongyloides* spp. were detected in any of the 131 samples. However, the prevalence of *Spirometra* spp. infection remained unchanged (Figure 3 and Table S2).

### 3.3. Growth and Development of Cat Hookworm, *Strongyloides* spp., and *T. cati* in Dry Conditions

In cat stool deposited on moist soil and nutrient agar, approximately 75%–80% of cat hookworm eggs could develop into L3. The rest were deformed. In contrast, for cat stool deposited on dry soil, approximately 20% of hookworm eggs could hatch within 24 h of incubation. The remaining 80% were immotile L1, deformed L1, embryonated eggs, and undivided eggs. Furthermore, we found some dead larva bodies, embryonated eggs, and undivided eggs at 48 h of incubation. After 80 h of incubation, we saw only deformed embryonated eggs and deformed undivided eggs. For *Strongyloides* spp., we found rare L3 and immotile free‐living female bodies with short buccal cavities at 24 h of incubation. At 60 h of incubation, we saw no larvae and free‐living worms. For *T. cati*, we found embryonated eggs after 2 weeks. The eggs could develop even in dry conditions (Table 7).

**TABLE 7:** Number of L3 of cat hookworm and *Strongyloides* spp. in stool placed on various media for 6 days and 6 weeks for a stool positive for *Toxocara cati*.

No. of	Stool deposited on		
	Dry soil	Wet soil	Nutrient agar
Hookworm L3 (*n* = 3)	0, 0, 0	412, 123, 364	455, 150, 322
*Strongyloides* spp. L3 (*n* = 3)	0, 0, 0	912, 541, 670	1120, 640, 760
*Toxocara cati* infective eggs (*n* = 1)	486	410	389

## 4. Discussion

Our previous study in humans revealed that the prevalence of STHs, including hookworm infection, trichuriasis, and ascariasis, decreased due to drought, STH control in the dry season, and improved sanitation and hygiene practices [21]. It is important to note that we cannot improve sanitation and hygiene in dogs and cats, and there is no anthelmintic administration; therefore, the El Niño 2014–2016‐induced drought was likely the factor that caused the decreased prevalence of hookworm infection in stray cats and dogs and eliminated toxocariasis and strongyloidiasis in stray dogs. However, other unmeasured factors, including changes in animal population dynamics, food availability, human activity, or environmental sanitation, may also have contributed to the observed reductions. According to our previous study in humans [21] and the present study in stray dogs and cats, the El Niño 2014–2016‐induced drought contributed to eliminating human ascariasis, dog toxocariasis, and strongyloidiasis and a decreased prevalence of human, dog, and cat hookworm infection, human trichuriasis, and human strongyloidiasis in southern Thailand, where anthelmintics are not applied.

During drought, the lack of rainwater carrying all stages, particularly L3, from one area to another area decreases the prevalence of hookworm infection [21, 25]. In southern Thailand, humans have been infected with *N. americanus* [26], while *Ancylostoma* spp., including *A. caninum*, *A. braziliense*, and *A. ceylanicum,* have infected dogs and cats. *N. americanus* L3 can enter the human host via skin penetration. In contrast, *Ancylostoma* spp. have several routes of infection by L3, including skin penetration, ingestion of the paratenic host, transplacental transfer, or transmammary from mother to fetus or infant [2]. Thus, *Ancylostoma* spp. L3 has infected a puppy or kitten since birth. This is why the prevalence of *N. americanus* infection in humans decreased, from 39.8% in 2013 to 8.6% in 2019 [21], more rapidly than that of ancylostomiasis in stray dogs from 70.5% in 2014 to 45.8% in 2020 (Table S2) and cats from 98.3% in 2014 to 60.3% in 2020 (Table S1), even though the life span of *N. americanus* is longer than *Ancylostoma* spp. [27].

L1 of *Strongyloides* spp. cannot complete the indirect cycle, producing L3, because of the dead free‐living males and females in dry stool (Table 7). However, the L1 can directly develop into L3 in 24 h before stool dryness [24]. Thus, the L3 has a greater chance of entering a new host than *Ancylostoma*. However, drought eliminates strongyloidiasis in dogs, not cats, possibly because of the different defecation habits. Dogs defecate on a street or soil surface, while cats defecate in a sandy hole they dig or on a sand surface covered loosely with sand. Dog stool, deposited on cement streets or dry soil, dries rapidly when exposed to sunlight and wind. A cat stool, deposited in a sandy hole covered by sand and prevented from sunlight exposure, may prevent water evaporation, resulting in extended survival of L3 of *Strongyloides* spp. from the direct cycle. In addition, shade under some trees covering a sandy beach prevents stool dryness. Combined with the ability to reproduce in the same host, the life cycle of *Strongyloides* spp. in cats could be maintained, resulting in the decrease in prevalence over time. Similarly, drought eliminated toxocariasis in dogs, not cats, due to the habit of defecating anywhere, especially on concrete streets and paved roads, where stool is directly exposed to sunlight all day. Although *Toxocara* eggs can develop into infective eggs in even dry stool, these eggs will die when exposed to sunlight and high temperatures (> 37°C) during drought (Table 1). Having similar eggshells to *A. lumbricoides* and *T. trichiura*, eggshells of *T. cati* and *T. canis* may shield the developing larvae inside from desiccation in the dry stool. However, eggshells cannot protect against high temperatures (> 37°C) [28]. Therefore, *Toxocara* eggs in dog stool cannot survive, but those in cat stool may survive due to the different defecation habits between dogs and cats. Cats defecate in sandholes, where some holes are under shades in the trees to protect the eggs from sunlight exposure. However, we have no data on direct behavioral or environmental measurements, including stool moisture content, sunlight exposure duration, or soil microclimate, to support the explanation of the different defecation habits between dogs and cats.

Lifecycles of STH in cats, including *Ancylostoma* hookworms, *T. cati,* and *Strongyloides* spp., were maintained in an unaffected area because cat stools were deposited in moist soil, where eggs or L1 could develop into L3, waiting to enter the new host. Therefore, the prevalence of STHs in cats in this area remained unchanged. In contrast, in the affected area, the life cycle of *Ancylostoma* hookworm in the dry environment was interrupted because egg and larval development ceased when cat stool was deposited in dry soil (Table 7). However, *Ancylostoma* L3 is capable of arresting in host tissue, and the following internal reinfection occurs, and the transmission of L3 to a kitten or puppy via milk or placental routes maintains the life cycle inside the host. Similarly, the life cycle of *Strongyloides* spp. was also interrupted. However, the prevalence of cat strongyloidiasis did not decrease because the ability to reproduce in the same host and shaded environment might protect L3 of *Strongyloides* spp. *T. cati* eggs can develop and survive in dry conditions in the shade under the trees in the affected area. Although the life span of *Toxocara* was expected to be equal to that of *A. lumbricoides* at only 1–2 years [27], it was not surprising that the prevalence of cat toxocariasis in the affected area without exposure to sunlight remained unchanged.

The prevalence of *Spirometra* spp. infection in cats in the unaffected area remained unchanged. Unfortunately, there was no *Spirometra* spp. in the affected area to compare. However, the unchanged prevalence of *Spirometra* spp. infection in dogs indicated that drought had no impact on the survival of this species due to its adult life of up to 9 years and its complex life cycle [13]. Water level in the reservoirs and the canals markedly decreased due to drought. However, the remaining water is enough to enhance the survival of copepods, the first intermediate host, and frogs, the second intermediate host, resulting in maintaining the life cycle of *Spirometra* spp. in the second study area. For stray cats in the unaffected area, the maintenance of *Spirometra* spp. might involve copepods attached to shrimps, crabs, and fish from the sea and some unknown second intermediate host.

La Niña 2017–2018 occurred between El Niño 2014–2016 and El Niño 2018–2019. In 2020, the prevalence of cat STHs in the affected area remained unchanged compared to 2016, despite the dry soil environment caused by drought in 2019 and 2020. It is possible that La Niña 2017–2018 induced heavy rainfall, resulting in moist soil, maintaining the life cycle of STH. Therefore, the prevalence of cat STHs in the affected area remained unchanged.

We did not know when El Niño would occur and how much drought would impact human and animal STH elimination. We just performed the STH control in humans. However, dogs and cats may act as reservoirs of some human STHs. Therefore, the animal’s stools must be examined. Accordingly, we also examined cat and dog stools coincidentally before and after the drought. So, the limitations are as follows: (1) We did not collect dog stools in 2016. The data may additionally enhance the data on cats in the affected area. (2) Unlike our previous study in humans (21), we do not collect data on each cat and dog to calculate metrics such as rates of reinfection, natural cure, and new infection. (3) We used microscopy alone. Molecular diagnostics, such as qPCR, would have offered greater sensitivity, particularly for detecting low‐intensity infections in the postdrought period, and could have confirmed the species of hookworms present. Future studies should incorporate these techniques. (4) While the consistent prevalence in the unaffected area over time strongly suggests stability, the small sample size (*n* = 17) limits the statistical power to detect minor fluctuations. (5) Limited geographic scope: The study is restricted to two locations in southern Thailand. Climatic, ecological, and cultural conditions in this region may not be generalizable to other tropical or subtropical settings.

## 5. Conclusions

In areas affected by drought, the prevalence of hookworm infection in cats and dogs decreased after the drought. Strongyloidiasis and toxocariasis in dogs were eliminated. The prevalence of strongyloidiasis in cats gradually decreased. However, there was no change in the prevalence of toxocariasis in cats, likely due to different defecation habits. However, the prevalence of *Spirometra* spp. infection remained unchanged. El Niño 2014–2016 may be a natural factor influencing a decrease in the prevalence of hookworm infection and strongyloidiasis in stray dogs and cats, but not in toxocariasis in stray cats. Climate forecasting should be integrated into future control strategies.

## Author Contributions

Prasit Na‐Ek designed and performed experiments, analyzed the data, and wrote the manuscript. Attarat Pattanawongsa and Patthanasak Khammaneechan conceived and designed experiments, analyzed the data, and wrote the manuscript. Blego Sedionoto analyzed the data and wrote the manuscript. Witthaya Anamnart conceived the study and writing–original draft. All authors discussed the results and reviewed the manuscript.

## Funding

No funding was received for this manuscript.

## Consent

The authors have nothing to report.

## Conflicts of Interest

The authors declare no conflicts of interest.

## Supporting Information

Additional supporting information can be found online in the Supporting Information section.

## Supporting information


**Supporting Information 1** Table S1 Supporting Table 1 Comparative prevalence and intensity of cat soil‐transmitted helminthiases and *Spirometra* spp. infection in stray cats between unaffected (artificial wet sandy soil) and affected (drought‐caused dry sandy soil) areas pre‐ and postdrought.

**Supporting Information 2** Table S2 Supporting Table 2 Comparative prevalence and intensity of soil‐transmitted helminthiases and *Spirometra* spp. infection in stray dogs pre‐ and postdrought in and around Walailak University.

## Data Availability

The data that support the findings of this study are available from the corresponding author upon reasonable request.
