# Supplementary material for: Impact of the 2014–2016 El Niño on Geohelminth Control in Stray Dogs and Cats Without Anthelmintic Treatment
Source: Vet Med Int. 2026 Jul 11;2026:9294504. doi: 10.1155/vmi/9294504 (PMC13355495; doi:10.1155/vmi/9294504)
Supplement: Supplementary file 1 — Supporting Information 1 Table S1 Supporting Table 1 Comparative prevalence and intensity of cat soil‐transmitted helminthiases and Spirometra spp. infection in stray cats between unaffected (artificial wet sandy soil) and affected (drought‐caused dry sandy soil) areas pre‐ and postdrought. [file VMI-2026-9294504-s002.docx]

| **Helminth** | **Hookworm** | ***Strongyloides* spp.** | ***Toxocara cati*** | ***Spirometra* spp.** |
| --- | --- | --- | --- | --- |
| **Unaffected area** |  |  |  |  |
| **Pre-drought 2014** (N=17) | **100%** (17/17; 95% CI: 81.6-100.0) | **47.1%** (8/17; 95% CI: 26.2-69.0) | **11.8%** (2/17; 95% CI: 3.3-34.3) | **11.8%** (2/17; 95% CI: 3.3-34.3) |
|  | 553 ± 333 (120-1200) | ND | 3400 ± 1131 (2600-4200) | ND |
| **Post-drought 2016** (N=18) | **100%** (18/18; 95% CI: 82.4-100.0) | **38.9%** (7/18; 95% CI: 20.3-61.4) | **11.1%** (2/18; 95% CI: 3.1-32.8) | **11.1%** (2/18; 95% CI: 3.1-32.8) |
|  | 492 ± 224 (100-1100) | ND | 4200 ± 849 (3600-4800) | ND |
| **2020**  (N=16) | **100%** (16/16; 95% CI: 80.6-100.0) | **31.3%** (5/16; 95% CI: 14.2-55.6) | **18.8%** (3/16; 95% CI: 6.6-43.0) | **12.5%** (2/16; 95% CI: 3.5-36.0) |
|  | 341 ± 189 (60-800) | ND | 4600 ± 2163 (2200-6400) | ND |
| **Affected area** |  |  |  |  |
| **Pre-drought 2014** (N=60) | **98.3%** (59/60; 95% CI: 91.1-99.7) ᵃ | **40.0%** (24/60; 95% CI: 28.6-52.6) | **11.7%** (7/60; 95% CI: 5.8-22.2) | **0%** (0/60; 95% CI: 0.0-6.0) |
|  | Med: 500 (IQR: 260-840) [60-1360] ᵇ | ND | 4557 ± 1624 (2600-7500) | ND |
| **Post-drought 2016** (N=60) | **58.3%** (35/60; 95% CI: 45.7-69.9) ᵃ | **23.3%** (14/60; 95% CI: 14.4-35.4) | **6.7%** (4/60; 95% CI: 2.6-15.9) | **0%** (0/60; 95% CI: 0.0-6.0) |
|  | Med: 260 (IQR: 120-520) [40-1020] ᵇ | ND | 2400 ± 548 (2000-3200) | ND |
| **2020**  (N=68) | **60.3%** (41/68; 95% CI: 48.4-71.1) | **19.1%** (13/68; 95% CI: 11.5-30.0) | **4.4%** (3/68; 95% CI: 1.5-12.2) | **0%** (0/68; 95% CI: 0.0-5.3) |
|  | Med: 260 (IQR: 150-560) [20-860] | ND | 953 ± 216 (800-1200) | ND |

**Supplementary Table 1** Comparative prevalence and intensity of cat soil-transmitted helminthiases and *Spirometra* spp. infection in stray cats between unaffected (artificial wet sandy soil) and affected (drought-caused dry sandy soil) areas pre- and post-drought.

Data are presented as prevalence % (n/N; 95% CI) and intensity. Intensity is presented as mean ± SD (range) for normally distributed data, and median (interquartile range, IQR) [range] for non-normally distributed data. ND = not done. ᵃ Fisher's exact test comparing 2014 and 2016 in the affected area: OR = 42.14 (95% CI: 5.47-324.77); *P* < .001. ᵇ Mann-Whitney U test comparing 2014 and 2016 in the affected area: Cliff's delta = 0.33; *P* = .007
