# Supplementary material for: Impact of the 2014–2016 El Niño on Geohelminth Control in Stray Dogs and Cats Without Anthelmintic Treatment
Source: Vet Med Int. 2026 Jul 11;2026:9294504. doi: 10.1155/vmi/9294504 (PMC13355495; doi:10.1155/vmi/9294504)
Supplement: Supplementary file 2 — Supporting Information 2 Table S2 Supporting Table 2 Comparative prevalence and intensity of soil‐transmitted helminthiases and Spirometra spp. infection in stray dogs pre‐ and postdrought in and around Walailak University. [file VMI-2026-9294504-s001.docx]

**Supplementary Table 2** Comparative prevalence and intensity of soil-transmitted helminthiases and *Spirometra* spp. infection in stray dogs pre- and post-drought in and around Walailak University.

| **Helminth** | **Hookworm in dog** | ***Strongyloides* spp.** | ***Toxocara canis*** | ***Spirometra* spp.** |
| --- | --- | --- | --- | --- |
| **Pre-drought** | | | | |
| 2014 (N=122) | **70.5**% (86/122; 95% CI: 61.9-77.9) ᵃ | **7.4%** (9/122; 95% CI: 3.9-13.4) ᶜ | **18.9%** (23/122; 95% CI: 12.9-26.7) ᵈ | **16.4**% (20/122; 95% CI: 10.9-24.0) |
|  | Med: 600 (IQR: 380-940) [60-4680] ᵇ | ND | 4539 ± 2158 (1200-9600) | ND |
| **Post-drought** | | | | |
| 2020 (N=131) | **45.8%** (60/131; 95% CI: 37.5-54.3) ᵃ | **0%** (0/131; 95% CI: 0.0-2.8) ᶜ | **0%** (0/131; 95% CI: 0.0-2.8) ᵈ | **10.7%** (14/131; 95% CI: 6.5-17.1) |
|  | Med: 100 (IQR: 40-310) [20-9800] ᵇ | ND | ND | ND |

Data are presented as prevalence % (n/N; 95% CI) and intensity. Intensity is presented as mean ± SD (range) for normally distributed data, and median (interquartile range, IQR) [range] for non-normally distributed data. ND = not done. ᵃ Pearson’s chi-square test: OR = 2.83 (95% CI: 1.68-4.75); *P* < .001. ᵇ Mann-Whitney U test: Cliff's delta = 0.58; *P* < .001. ᶜ Fisher’s exact test: *P* = .001. ᵈ Fisher’s exact test: *P* < .001.
